# Association between serum uric acid levels and diabetic peripheral neuropathy in type 2 diabetes: a systematic review and meta-analysis

**DOI:** 10.3389/fendo.2024.1416311

**Published:** 2024-07-12

**Authors:** Xieyu Zhang, Xinwen Zhang, Xiaoxu Li, Xin Zhao, Guangcheng Wei, Jinjie Shi, Yue Yang, Su Fan, Jiahe Zhao, Ke Zhu, Jieyang Du, Junyi Guo, Wei Cao

**Affiliations:** ^1^ Department of Rheumatology, Wangjing Hospital, China Academy of Chinese Medicine Science, Beijing, China; ^2^ Robotics Movement Department, Amazon, Boston, MA, United States

**Keywords:** meta-analysis, systematic review, diabetic peripheral neuropathy, hyperuricemia, uric acid

## Abstract

**Background:**

The evidence supporting a connection between elevated serum uric acid (SUA) levels and diabetic peripheral neuropathy (DPN) is controversial. The present study performed a comprehensive evaluation of this correlation by conducting a systematic review and meta-analysis of relevant research.

**Method:**

PubMed, Web of Science (WOS), Embase, and the Cochrane Library were searched for published literature from the establishment of each database to January 8, 2024. In total, 5 cohort studies and 15 cross-sectional studies were included, and 2 researchers independently screened and extracted relevant data. R 4.3.0 was used to evaluate the included literature. The present meta-analysis evaluated the relationship between SUA levels and the risk of DPN in type 2 diabetes (T2DM) by calculating the ratio of means (RoM) and 95% confidence intervals (CIs) using the method reported by JO Friedrich, and it also analyzed continuous outcome measures using standardized mean differences (SMDs) and 95% CIs to compare SUA levels between DPN and non-DPN groups. Funnel plot and Egger’s test were used to assess publication bias. Sensitivity analysis was conducted by sequentially removing each study one-by-one.

**Results:**

The meta-analysis included 20 studies, with 12,952 T2DM patients with DPN and 16,246 T2DM patients without DPN. There was a significant correlation between SUA levels and the risk of developing DPN [odds ratio (OR) = 1.23; 95% CI: 1.07-1.41; p = 0.001]. Additionally, individuals with DPN had higher levels of SUA compared to those without DPN (SMD = 0.4; 95% CI: -0.11-0.91; *p* < 0.01).

**Conclusion:**

T2DM patients with DPN have significantly elevated SUA levels, which correlate with a heightened risk of peripheral neuropathy. Hyperuricemia (HUA) may be a risk indicator for assessing the risk of developing DPN in T2DM patients.

**Systematic review registration:**

https://www.crd.york.ac.uk/PROSPERO, identifier CRD42024500373.

## 1 Introduction

Serum uric acid (SUA) is the product of purine catalysis by xanthine oxidase 1 (1). Hyperuricemia (HUA), a metabolic disorder related to purine metabolism, is increasingly becoming a significant global health concern. HUA was initially primarily recognized for its association with gout and the resulting impact on quality of life (2). However, recent research has provided evidence linking HUA to various other conditions, such as cardiovascular diseases, renal dysfunction, and cancers (3–5). Previous studies have suggested a high prevalence of HUA among type 2 diabetes mellitus (T2DM) patients (6–10). HUA in diabetic patients can be attributed to a range of factors, such as elevated body mass, larger waist circumference, abnormal lipid levels, lack of physical activity, high blood pressure, and insulin resistance (11, 12), resulting in an unfavorable prognosis and an increase in complications associated with diabetes, such as neuropathy, retinopathy, and nephropathy (13).

The prevalence of diabetes-related complications is increasing worldwide. The influence of uric acid levels on the development of vascular complications has been assessed in individuals diagnosed with DM (14), and the correlation between uric acid levels and complications related to diabetes mellitus (DM) has garnered significant interest. Diabetic peripheral neuropathy (DPN) is a long-term complication linked to DM, with a prevalence ranging from 60% to 90%. Approximately half of patients do not exhibit any symptoms (15), and there is a significant incidence of disability and mortality associated with this condition (16). DPN is a condition of irreversible damage to the nerves, resulting in a gradual decline in sensory function starting from the lower limbs. DPN is further distinguished by significant morbidity and is accompanied by pain (17). There is an incomplete understanding of the factors contributing to the formation of DPN, but numerous hypotheses have proposed a multifactorial mechanism (18) involving various factors, such as elevated condensation levels, length of diabetes diagnosis, presence of high blood pressure, tobacco use, alcohol consumption, excessive weight gain, and HUA (19–22). Because the impact of uric acid on the progression of DPN is unknown, it is necessary to investigate the correlation between uric acid and DPN. There is currently a lack of effective treatments available for DPN that can reverse neuronal damage. Symptom management, such as pain relief, is primarily achieved through pharmacotherapy; however, this approach improves quality of life for some patients but is not effective for all (23–28). Early screening of risk factors will help explore new therapeutic approaches for DPN (27). Considering that SUA levels can be modified, interventions aimed at reducing uric acid may potentially serve as preventive or treatment strategies for DPN in individuals with DM.

The correlation between HUA and neurological disorders is two-sided, in which both low and high SUA may have adverse effects. Given the crucial role of SUA as an antioxidant, maintaining excessively low levels of SUA for an extended period may potentially expose individuals with DM to increased oxidative stress (OS) and disorders related to nerve damage (29). Conversely, HUA may facilitate the movement of smooth muscle cells into blood vessels and block the release of nitric oxide (NO) by endothelial cells, which may result in impaired blood vessel performance, inadequate blood supply to tissues, and impaired functionality of nerves in the peripheral region (30). Multiple studies have suggested a relationship between HUA concentrations and the heightened prevalence of DPN (31–33). A meta-analysis published in 2016 has indicated that patients diagnosed with DM accompanied by DPN exhibit noticeable increases in UA levels and that the presence of HUA is linked to an augmented risk of developing peripheral neuropathy (8). After 2016, additional cross-sectional studies, case-control studies, and other large-sample studies have been published, and some of the results are inconsistent with those of previous studies. For example, a population-based cross-sectional study in China has reported that patients with a lower level of uric acid have a higher risk of DPN compared to those with a normal level of uric acid, disagreeing with earlier findings (34). Because the evidence supporting the correlation between elevated SUA levels and DPN is controversial, the present study analyzed relevant studies to provide a comprehensive evaluation of this correlation to update and supplement existing research.

## 2 Materials and methods

### 2.1 Study registration

The present study was conducted following the recommendations of the Preferred Reporting Items for Systematic Reviews and Meta-Analyses (PRISMA). The PRISMA checklist was presented as **Supplementary Material 1**. Additionally, the present protocol for the systematic review and meta-analysis was registered with the Prospective Register of Systematic Reviews (PROSPERO; registration number CRD42024500373).

### 2.2 Search strategy

A comprehensive systematic search was conducted using the following four electronic databases: PubMed, Web of Science (WOS), Embase, and the Cochrane Library. This search spanned from the inception of each database to January 8, 2024. The following keywords were used to search the databases: uric acid or urate or hyperuricemia and diabetes and neuropathy or peripheral neuropathy. The complete search strategy is presented in **Supplementary Material 2**. The references of the included studies were further examined to identify additional relevant papers. Relevant systematic review or meta-analysis studies were identified and evaluated for inclusion into the present analysis.

### 2.3 Selection criteria

The inclusion criteria for the meta-analysis were as follows: (1) cohort, cross-sectional, or case-control studies; (2) examined the potential association between SUA levels and DPN among individuals with T2DM or compared SUA levels in patients with DPN versus a control group without DPN; (3) control groups consisted of T2DM patients without neuropathy; (4) utilized accurate and precise methodologies for uric acid level measurement; and (5) provided relevant data applicable for meta-analysis. The exclusion criteria were as follows: reviews, letters to the editor, conference papers, editorials, comments, case reports, and any articles not available in full text. Following the systematic search, two researchers (Xinwen Zhang and Xieyu Zhang) assessed the eligibility of the studies based on the titles and abstracts of all identified records. In case of any disagreement during the assessment process, a third researcher, Xiaoxu Li, was consulted to help reach a consensus.

### 2.4 Data extraction

For each included study, two investigators (Xinwen Zhang and Xieyu Zhang) independently extracted the following information using a predefined data extraction form: first author, publication year, study design, country of study, sample size, age (mean and standard deviation), gender, SUA levels (mean and standard deviation), odds ratio (OR) estimates, corresponding 95% confidence intervals (Cis), and matched or adjusted factors. OR estimates were extracted from the most comprehensively adjusted model in each study, aiming to minimize the impact of non-measured confounding factors.

To standardize the analysis, SUA levels reported in mg/dL were uniformly converted to μmol/L using the following conversion formula: 1 mg/dL = 59.48 μmol/L. If the levels of SUA were presented as medians and interquartile ranges, they were converted to means and standard deviations (35). All discrepancies were solved by a third investigator (Xiaoxu Li).

### 2.5 Risk of bias assessment

The risk of bias in the included articles was assessed by two reviewers (Xinwen Zhang and Xieyu Zhang) utilizing two distinct tools. The analytical cross-sectional studies meeting the inclusion criteria were assessed using the Joanna Briggs Institute (JBI) checklist (36). The JBI checklist was scored on a scale from 0 to 8, with each “yes” response to questions receiving 1 point. Conversely, responses marked as “no” or “unclear” were assigned 0 points. Cross-sectional studies achieving scores of 5 or higher on this scale were categorized as high quality. The included cohort and case-control studies were evaluated for the quality of selection, comparability, and outcome based on the Newcastle-Ottawa Quality Assessment Scale (NOQAS). The NOQAS scores ranged from 0 (poor) to 9 (excellent), with a score of 6 or higher indicating high quality studies. In instances of disagreement during the assessment process, a third researcher (Xiaoxu Li) was consulted to reach consensus.

### 2.6 Data synthesis and analysis

Meta-analyses were conducted using R 4.3.0 (R Foundation for Statistical Computing, Vienna, Austria; https://www.R-project.org/). To facilitate a more effective analysis and comparison of studies reporting different types of outcomes (continuous and binary), the present meta-analysis was divided into two main parts. First, the relationship between SUA levels and the risk of DPN in T2DM patients was assessed using ORs and 95% CIs. Among the included studies, some studies reported continuous outcomes. For these studies, the method proposed by JO Friedrich was used to calculate the ratio of means (RoM) and 95% CIs (37), which were then integrated into the broader analysis. Second, to compare SUA levels between T2DM patients with DPN and those without DPN, studies reporting continuous outcome measures were analyzed using standardized mean differences (SMDs) and 95% CIs in a meta-analytic approach.

Random effects models were used based on the assumption that there was true heterogeneity among studies due to variations in populations and settings where the studies were conducted. Heterogeneity in outcomes was assessed through multiple methods. Although it may be overly sensitive in meta-analyses encompassing a large number of studies, Cochran’s Q test and its associated p-value were reported (38). Furthermore, the I^2^ statistic, which quantifies the percentage of variance attributable to true effect differences rather than sampling error (39), was also reported. Given that meta-analyses of prevalence often result in elevated I^2^ values, which may not accurately reflect true heterogeneity, prediction intervals were additionally presented (40). These intervals forecast the expected range of outcomes in 95% of comparable studies, thereby elucidating the extent of uncertainty in the estimated outcomes (41). Additionally, to account for the impact of variables, such as study design, study region, and sample size, on heterogeneity within the study literature, subgroup analyses were conducted. The potential publication bias was assessed by a funnel plot and Egger’s test. Sensitivity analysis was performed by removing each study one-by-one to verify the robustness of the pooled value.

## 3 Results

### 3.1 Selection of studies

**Figure 1:**
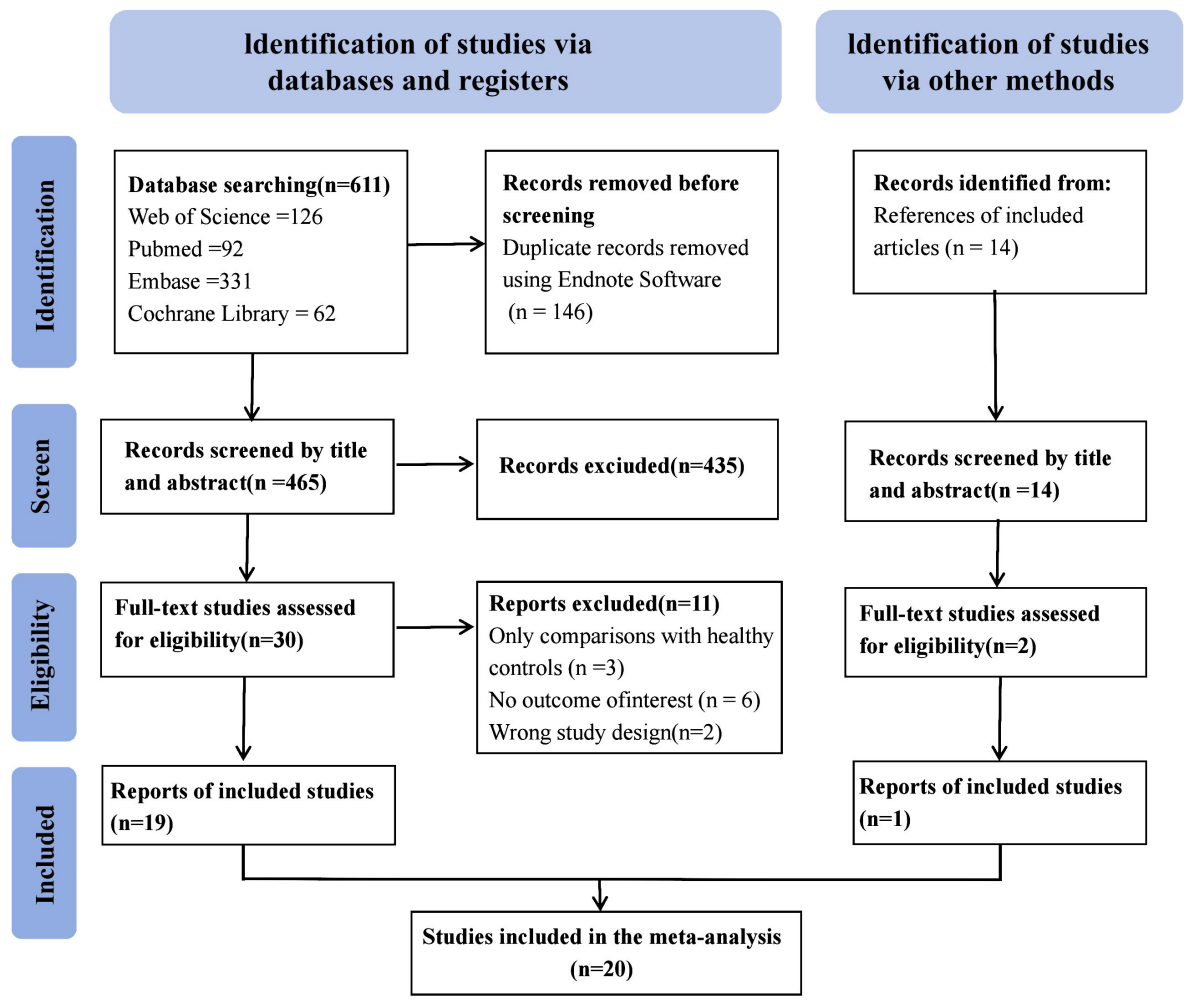
Flow chart of the study selection process.

The electronic search of 4 databases yielded 549 studies. Additionally, a manual review of the references from these studies identified one additional eligible study, resulting in 550 studies. Ultimately, 20 articles met the inclusion criteria for the present meta-analysis. The search process is summarized in **Figure 1**.

### 3.2 Basic characteristics of the included studies

**Table 1** shows the essential characteristics of the 20 articles incorporated into the present meta-analysis. The present analysis comprised 29,198 patients, including 12,952 patients diagnosed with DPN and 16,246 patients without DPN. Moreover, there were 15 cross-sectional studies and 5 cohort studies. The included literature originated from the following locations: nine articles from China; two articles from Iran and Thailand; and one article from the United States, Canada, India, Indonesia, Pakistan, Greece, and Australia.

**Table 1:** Basic characteristics of included literature.

Author	Year	Country	Study design	Sample Size (cases/controls)	Age (cases/controls)	Gender(female/male)		Outcomes	Matched factors or adjusted factors	Quality
						cases	controls			
Han Y et al.(42)	2023	America	Cross-sectional study	403/1050	–	–	–	OR	poverty–income ratio, waist circumference, smoking status, education level, hypertension, serum triglyceride, total cholesterol, courses of diabetes (year), HbA1c	8
Uzeli U et al.(43)	2023	China	Case-control study	50/50	59.7 ± 7.9/56.6 ± 9.8	25/25	34/16	Mean ± SD	None	6
Wang W et al.(34)	2023	China	Cross-sectional study	10084/4824	62.6 ± 12.5/58.5± 13.5	4365/5719	1957/2867	Mean ± SD	None	7
Zhang H et al.(32)	2023	China	Cross-sectional study	57/49	56.0 ± 13.7/55.4 ± 10.6	23/34	17/32	OR	None	6
Zhang J et al.(44)	2023	China	Cross-sectional study	34/29	51.6 ± 13.1/52.9 ± 7.6	11/23	10/19	Mean ± SD	None	6
Zhuang Y et al.(31)	2022	China	Cross-sectional study	150/250	50.7 ± 7.8/51.0 ± 8.6	69/81	116/134	Mean ± SD; OR	disease course, age, HbA1c, diabetic retinopathy(%), eGFR, Vit B_12_	7
Fayazi HS et al.(45)	2022	Iran	Case-control study	115/115	–	78/37	83/33	Mean ± SD; OR	gender, systolic blood pressure, diabetic retinopathy, hypertension history, HbA1c, microalbuminuria,	8
Zhang W et al.(46)	2022	China	Cross-sectional study	471/176	59.3 ± 12.2/51.3 ± 13.5	173/301	73/103	Mean ± SD; OR	age, sex, duration of diabetes, HbA1c, body mass index, smoking, hypertension, total cholesterol, triglyceride, high-density lipoprotein cholesterol, low-density lipoprotein cholesterol, and free thyroxine (FT4)	8
Wu B et al.(47)	2021	China	Cross-sectional study	219/393	–	–	–	OR	None	6
Kaewput W et al.(48)	2020	Thailand	Cross-sectional study	226/7285	–	–	–	OR	age, gender, duration of T2DM, hypertension, dyslipidemia, coronary artery disease, cerebrovascular disease, diabetic retinopathy, smoking, insulin, renin angiotensin aldosterone system blockade, antiplatelet, statins, body mass index, fasting plasma glucose level, estimated glomerular filtration rate	7
Jiang TN et al.(49)	2019	China	Cross-sectional study	503/321	59.8 ± 9.5/46.1 ± 11.8	253/250	110/211	Mean ± SD; OR	None	6
Lin X et al.(19)	2018	China	Cross-sectional study	77/123	60.8 ± 13.4/52.8 ± 11.1	–	–	Mean ± SD; OR	age, sex, smoking history, duration of diabetes, systolic blood pressure, diastolic blood pressure, body mass index, HbA1c, triglycerides, total cholesterol, low-density lipoprotein, high-density lipoprotein	7
Ranjith KP et al.(50)	2018	India	Cross-sectional study	55/63	55.6± 18.3/51.6 ± 20.5	20/35;	29/34	Mean ± SD	None	7
Sukarno DP et al.(51)	2018	Indonesia	Case-control study	15/15	51.60 ± 6.52/53.53 ± 4.72	10/5;	10/5	OR	HbA1c, disease duration	6
Abraham A et al.(52)	2017	Canada	Case-control study	115/38	62.0 ± 13.0/61.0 ± 9.0	45/70;	20/18	Mean ± SD	None	6
Dar UF et al.(53)	2016	Pakistan	Cross-sectional study	88/112	–	35/53;	44/68	Mean ± SD	None	6
Kiani J et al.(54)	2013	Iran	Case-control study	42/42	54.6 ± 6.9/55.8 ± 5.8	26/16;	26/16	Mean ± SD	None	6
Chuengsamarn S et al.(55)	2014	Thailand	Cross-sectional study	102/506	–	–	–	OR	Modification of diet in renal disease-glomerular filtration rate, smoking, statin user, angiotensin-converting enzyme inhibitors user, angiotensin II receptor blockers user, microalbuminuria	7
Papanas N et al.(56)	2011	Greece	Cross-sectional study	64/66	63.0 ± 2.8/62.4 ± 3.1	33/31;	34/32	Mean ± SD	None	6
Tapp RJ et al.(57)	2003	Australia	Cross-sectional study	82/739	73.0 ± 10/62.0 ± 12.0	37/45;	362/377	Mean ± SD; OR	Age, diabetes duration, height	7

### 3.3 Evaluation of the methodological quality of the studies

The JBI checklist scores for the included cross-sectional studies varied from 6 to 8, while the NOQAS scores for the included case-control studies ranged from 6 to 8. These scores indicated that the quality of the studies was medium to high.

### 3.4 Meta-analysis of SUA levels and DPN risk in T2DM patients

**Figure 2:**
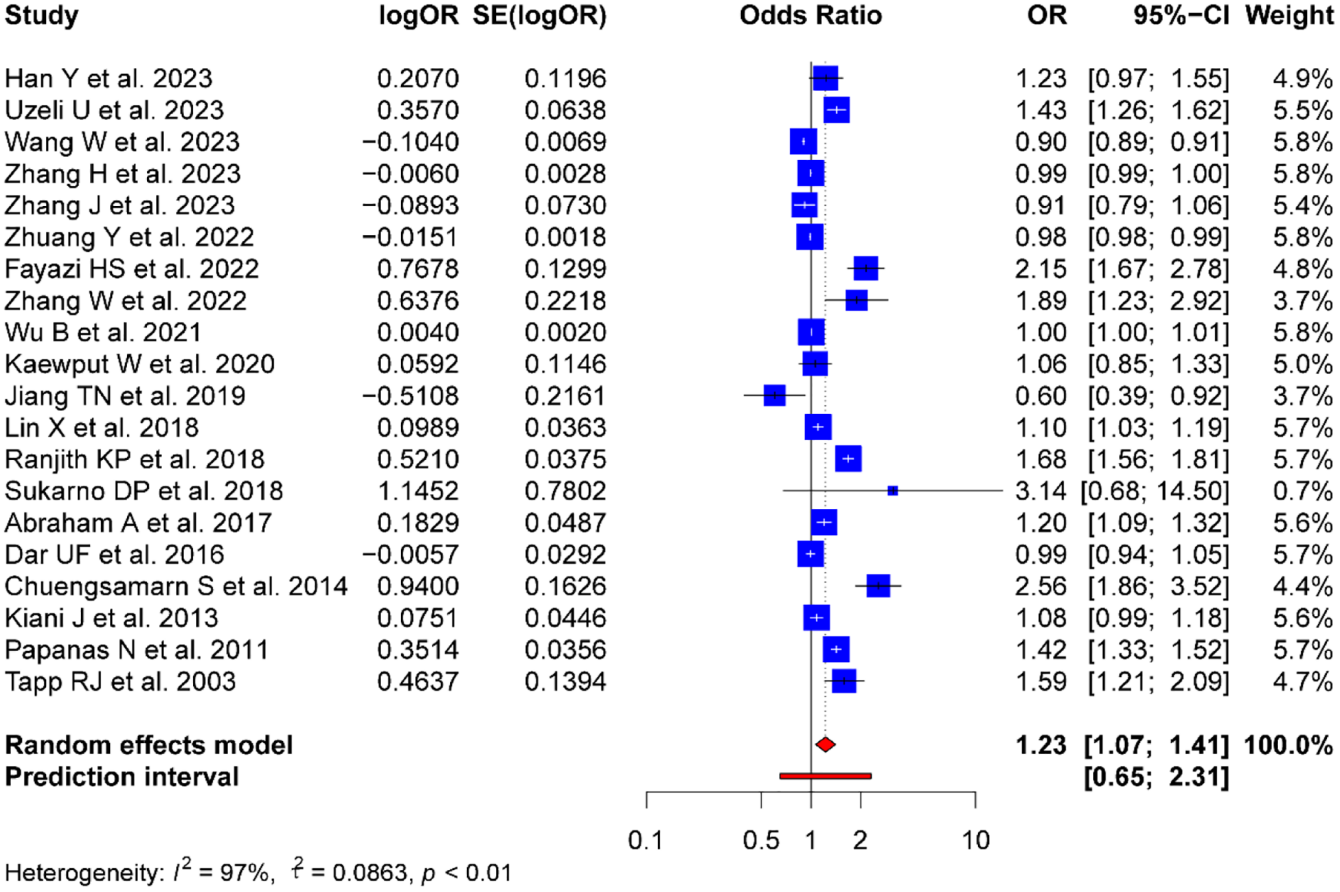
Forest plot evaluating the association between SUA level and DPN risk.

The meta-analysis of SUA levels and the risk of DPN yielded a combined effect size OR of 1.23 (95% CI: 1.07-1.41; Prl: 0.65-2.31). Moreover, the heterogeneity of the included studies was high (p = 0.001, I^2^ = 97%, τ2 = 0.0863) (**Figure 2**).

#### 3.4.1 Subgroup analysis

To further explore the sources of heterogeneity of the relationship between SUA levels and the risk of DPN in T2DM patients, subgroup analyses based on study type, country, and sample size were conducted.

Among the 15 cross-sectional studies, the combined OR was 1.17 (95% CI: 1.00-1.38), with significant heterogeneity (p < 0.01, I^2^ = 98%, τ2 = 0.0890). In the five case-control studies, the combined OR was 1.23 (95% CI: 1.07-1.41), also showing substantial heterogeneity (p < 0.01, I^2^ = 88%, τ2 = 0.076).

Subgroup analysis by country revealed the following outcomes: the combined OR for the nine Chinese studies was 1.04 (95% CI: 0.9-1.19, p < 0.01, I^2^ = 96%, τ2 = 0.0374); the combined OR for the Iranian studies (n=2) was 1.51 (95% CI: 0.77-2.97, p < 0.01, I^2^ = 95%, τ2 = 0.2305); and the combined OR for the two Thai studies was 1.64 (95% CI: 0.69-3.88, p < 0.01, I^2^ = 95%, τ2 = 0.3681). The studies from the other regions were insufficient for subgroup analysis.

A subgroup analysis was conducted based on sample size. The quartile method was used to divide the studies into four groups according to their sample sizes, ensuring that each group contained a relatively uniform amount of data to reduce the impact of subjective division. The following four groups based on sample size were analyzed: Group 1 (Q1), which included sample sizes less than 115; Group 2 (Q2), which included sample sizes between 115 and 215; Group 3 (Q3), which included sample sizes between 215 and 718; and Group 4 (Q4), which included sample sizes greater than 718. Meta-analyses were conducted for each sample size group, yielding the following results: Q1: OR = 1.40 (95% CI: 0.91-2.14, p < 0.01, I^2^ = 94%, τ2 = 0.1926); Q2: OR = 1.26 (95% CI: 1.05-1.51, p < 0.01, I^2^ = 97%, τ2 = 0.0426); Q3: OR = 1.30 (95% CI: 0.94-1.79, p < 0.01, I^2^ = 96%, τ2 = 0.1223); and Q4: OR = 1.04 (95% CI: 0.79-1.38, p < 0.01, I^2^ = 86%, τ2 = 0.0866). Based on the analysis results of these four groups, the sample size impacted the effect size and heterogeneity. The groups with smaller sample sizes (Q1 and Q2) showed larger effect sizes and heterogeneity, which may be related to the instability and more significant variability of results in studies with small sample sizes. The groups with larger sample sizes (Q3 and Q4) exhibited smaller effect sizes that were not statistically significant and slightly lower heterogeneity. The forest plots for these subgroup analyses are shown in **Supplementary Material 3**.

Despite conducting subgroup analyses, there was still persistent high heterogeneity across the groups, which suggested that the included factors, such as study type, country, and sample size, may not fully explain the variability observed in the relationship between uric acid levels and the risk of DPN in T2DM patients. Thus, other factors that affect heterogeneity may be involved, including differences in experimental conditions (such as the stage of DPN among participants), demographic variations (such as age and gender), varying diagnostic criteria, and different measurement methods for determining uric acid levels and DPN status.

#### 3.4.2 Sensitivity analysis

**Figure 3:**
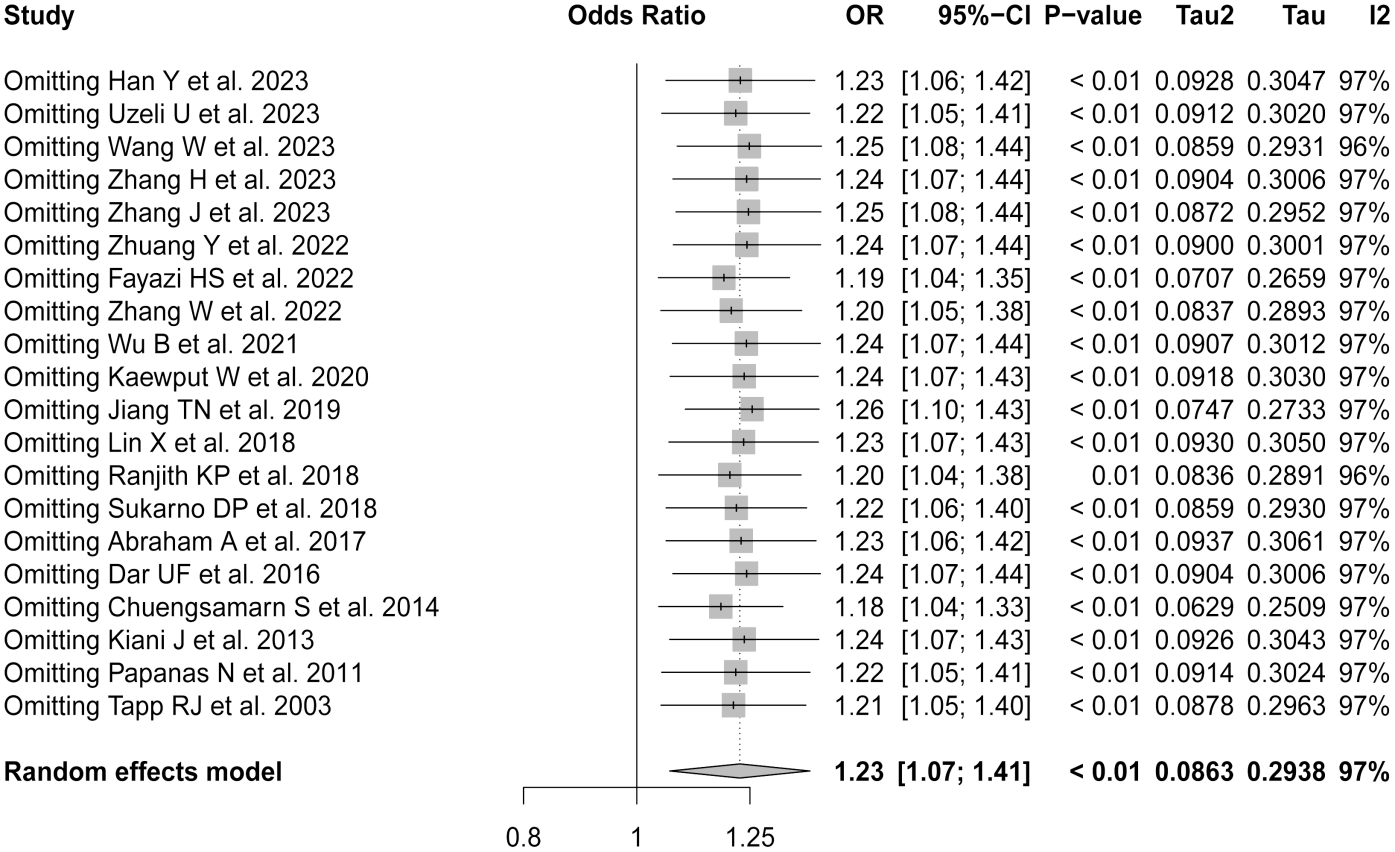
Forest plot of sensitivity analysis by sequentially removing each study.

**Figure 3** illustrates the robustness of the present analyses. For the sensitivity analysis, each study was sequentially excluded from the meta-analysis to assess the impact on the overall results. Excluding individual studies did not significantly alter the combined ORs. This lack of substantial variation in the outcomes with the exclusion of each study validated the stability and reliability of the present findings. Despite the high heterogeneity observed in the primary analysis, these results confirmed the reported associations between uric acid levels and the risk of DPN in T2DM patients, supporting their relevance in the broader context of diabetes research.

#### 3.4.3 Publication bias

The contour-enhanced funnel plot for the meta-analysis suggested no evidence of publication bias. In addition, the Begg’s test (p = 0.7952) and Egger’ test (p=0.0552) results were not statistically significant, indicating no publication bias (**Supplementary Material 4**).

### 3.5 Comparison of SUA levels between DPN and non-DPN patients

**Figure 4:**
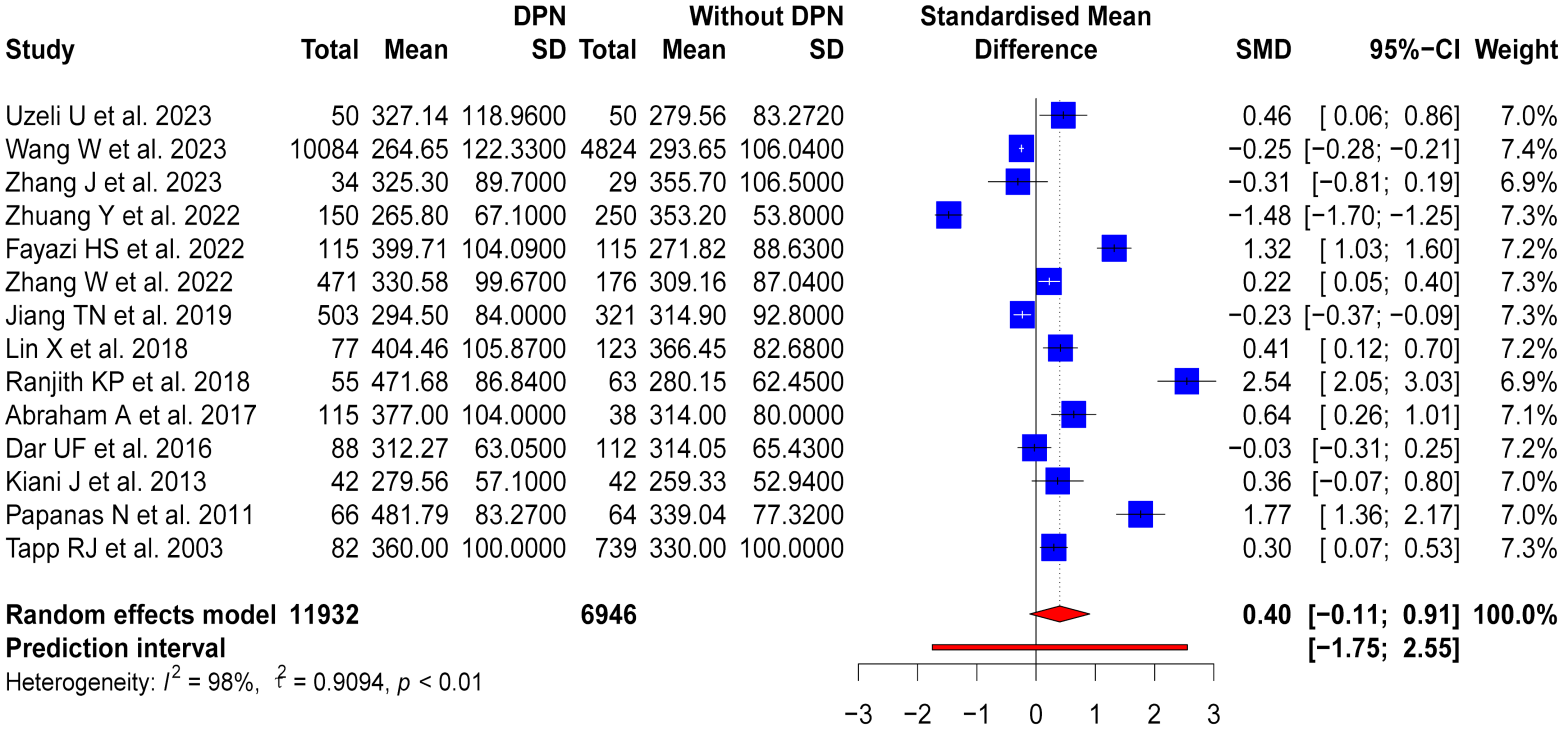
SUA levels in DPN patients compared to those without DPN.

Fourteen studies compared SUA levels between DPN patients and non-DPN patients, which included 11,932 DPN patients and 6,946 non-DPN patients. DPN patients had higher SUA levels than non-DPN patients, with an SMD of 0.4 (95% CI: -0.11-0.91; Prl: -1.75-2.55). However, high heterogeneity was observed in these studies (p < 0.01, I^2^ = 98%, τ2 = 0.9094) (**Figure 4**).

#### 3.5.1 Subgroup analysis

To investigate factors contributing to variability, subgroup analyses based on study design, country of study, and sample size were conducted. For the ten cross-sectional studies, a moderate variation in SUA levels was observed between DPN and non-DPN patients (SMD = 0.28, 95% CI: -0.4-0.97), but there was high heterogeneity among these studies (p < 0.01, I^2^ = 98%, τ2 = 1.2096). The four case-control studies showed a more noticeable difference in SUA levels (SMD = 0.71, 95% CI: 0.27-1.15), but there was also significant heterogeneity (p < 0.01, I^2^ = 85%, τ2 = 0.1651). Moreover, notable geographical variations were observed in the subgroup analyses based on the country of study. The seven studies from China revealed a slight reduction in SUA levels in patients with DPN (SMD = -0.17, 95% CI: -0.66-0.32), but there was considerable heterogeneity (p < 0.01, I^2^ = 97%, τ2 = 0.4212). Conversely, the two Iranian studies indicated an increase in SUA levels in patients with DPN (SMD = 0.86, 95% CI: -0.08-1.79), but there was high heterogeneity (p < 0.01, I^2^ = 92%, τ2 = 0.4207). Further subgroup analysis was conducted based on the sample size, dividing the studies into four groups using quartiles. The first group (Q1) had a sample size of less than 165, the second group (Q2) had a sample size between 165 and 315, the third group (Q3) had a sample size between 315 and 778, and the fourth group (Q4) had a sample size greater than 778. The results of the subgroup analysis showed that the group with the smallest sample size, Q1, had an OR of 1.16 (95% CI: -0.06-2.38, p < 0.01, I2 = 96%, τ2 = 1.5005), Q2 had an OR of 0.57 (95% CI: -0.21-1.34, p < 0.01, I2 = 96%, τ2 = 0.4507), Q3 had an OR of -0.30 (95% CI: -1.46-0.86, p < 0.01, I2 = 99%, τ2 = 1.0308), and Q4 had an OR of 0.04 (95% CI: -0.31-0.38, p < 0.01, I2 = 91%, τ2 = 0.1113). These results suggest that the groups with smaller sample sizes (Q1 and Q2) exhibited higher heterogeneity and more significant effect sizes, but the results were unstable and not statistically significant. In contrast, the groups with larger sample sizes (Q3 and Q4) showed smaller effect sizes but still had some degree of heterogeneity. These results highlighted the diverse impacts of HUA on patients with DPN, which were influenced by study design, geographical location, and sample size, thereby warranting further investigation. The forest plots for these subgroup analyses are shown in **Supplementary Material 5**.

#### 3.5.2 Sensitivity analysis

**Figure 5:**
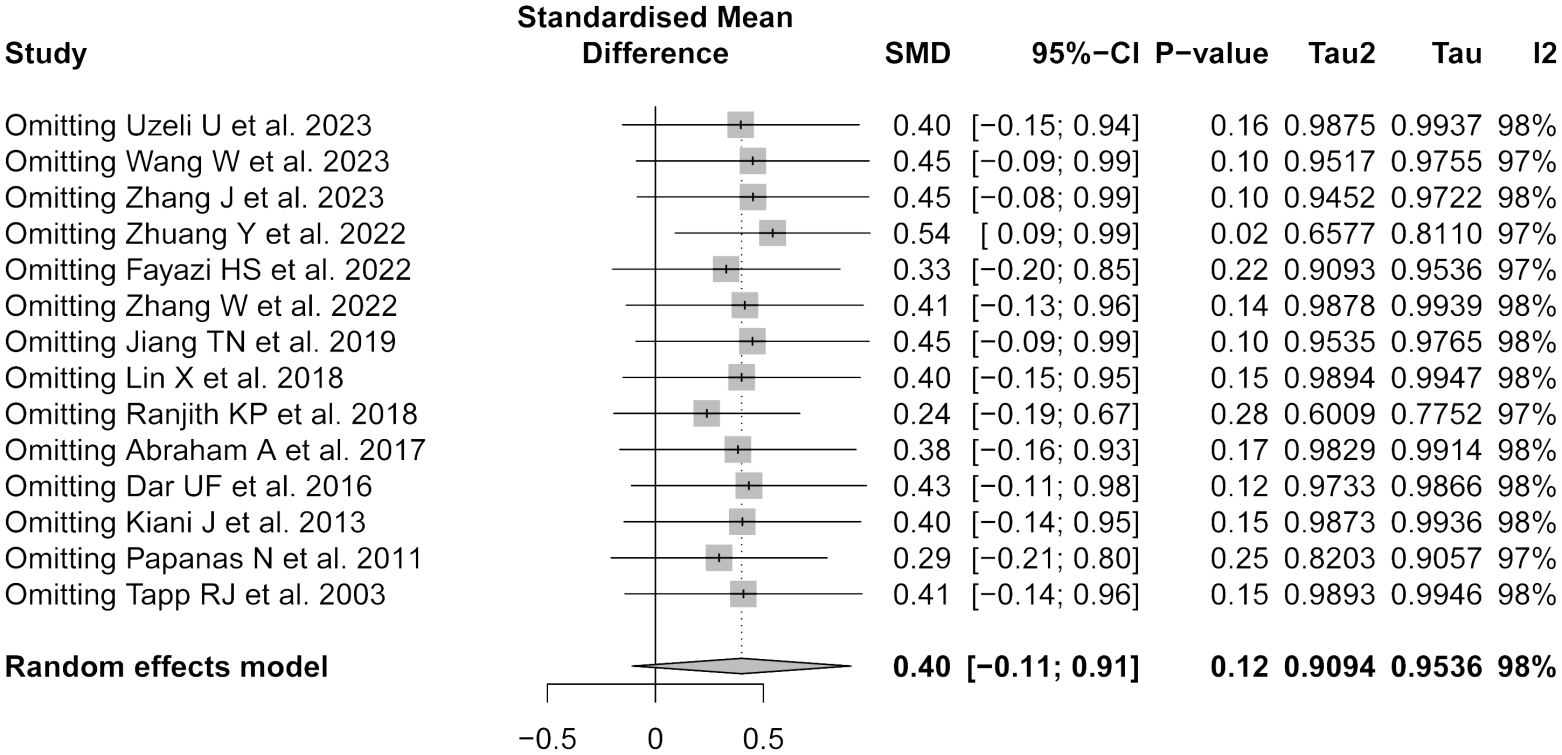
Forest plot of sensitivity analysis by sequentially removing each study.

The sensitivity analysis confirmed the robustness of the meta-analysis (**Figure 5**). Exclusion of any single study did not significantly alter the overall effect size, underscoring the stability of the present findings.

#### 3.5.3 Publication bias

A contour-enhanced funnel plot was utilized alongside Egger’s and Begg’s tests to assess publication bias in the present meta-analysis. The funnel plot was asymmetrical as indicated by the dispersion of studies outside the central area of the funnel (**Supplementary Material 6**). In addition, Egger’s test yielded a p-value of 0.0388, suggesting potential publication bias, but Begg’s test showed a p-value of 0.208, indicating no significant evidence of bias. The discrepancy between these tests may be attributed to the greater sensitivity of Egger’s tests to detect bias, especially in studies with smaller sample sizes. Overall, these findings suggested that the present results should be interpreted with caution, taking into account the possibility of publication bias, as suggested by Egger’s test.

## 4 Discussion

Until now, a comprehensive analysis of the association between HUA and DPN in individuals diagnosed with DM has been lacking. The present study performed a systematic review and meta-analysis, which was comprised of 20 studies, including 12,952 T2DM patients with DPN and 16,246 T2DM patients without DPN, to thoroughly examine the relationship between HUA and DPN development.

DPN is a severe and long-term complication, resulting from prolonged high blood sugar, which damages peripheral nerves. Approximately 50% of DM patients may experience neuropathy in their lifetime (58). Cross-sectional and cohort studies conducted since 2016 have reported a DPN incidence of approximately 8.8/1,000 person-years among individuals with type 1 diabetes mellitus (T1DM) (59) and 24–26.9/1,000 person-years among individuals with T2DM (59, 60). DPN prevalence is generally 30% (61, 62). A recent worldwide meta-analysis (29 studies with 50,112 participants) has reported that individuals with T2DM have higher DPN prevalence (31.5%, 95% CI 24.4–38.6%) compared to those with T1DM (17.5%, 95% CI 13.1–36.5%) (63). DPN is characterized by neuropathic pain, numbness, and sensory abnormality on symmetrical, bilateral distal limbs, which not only increases the risks of foot ulceration and even lower limb amputation but also affects the patient’s health, thereby causing a heavy financial burden (17). Moreover, DPN may heighten cardiovascular disease risks (17). Diabetes duration is a strong DPN determinant, and DPN prevalence varies by country and ranges from 1% to 80% (64). This large variation likely arises from multiple factors, including disease severity, diabetes duration, DPN definition, and comorbid conditions predisposing to neuropathy development, especially metabolic syndrome. Nerve conduction is often used as a standard diagnostic tool for DPN, but it is time-consuming, costly, and somewhat difficult to clinically diagnose (65, 66). As a metabolic disease, the occurrence of DPN has been suggested to be related to the imbalance of metabolic pathways caused by hyperglycemia, lipid metabolism disorders, and insulin abnormalities, which can lead to OS, inflammatory reaction, mitochondrial dysfunction, and nerve cell damage (67). Because there is no effective treatment for DPN, early glycemic control combined with exercise and a healthy diet is suggested to prevent and delay disease progression (26). The potential pathogenic mechanisms of DPN remain unclear, limiting the exploration of useful prevention and treatment strategies for DPN (65, 66).

Elevated SUA levels are typically characterized by SUA levels greater than 6.8 mg/dL, indicating a higher likelihood of developing gout. Earlier research has indicated a potential association between elevated levels of uric acid in the blood and the development of cardiovascular conditions (68, 69). Studies have suggested that there is a potential association between HUA and several conditions, such as elevated glucose levels, reduced insulin sensitivity, irregular lipid profiles, and metabolic syndrome (70–75), which may contribute to the onset of diabetic neuropathy (76). Previous studies have suggested a high prevalence of HUA among T2DM patients (6–10), and there is an association between levels of uric acid and DPN, suggesting that uric acid may serve as an indicator for HUA-induced OS in the progression of diabetic neuropathy (77–80). Once inside cells, uric acid activates specific mitogen-activated protein kinases (MAPKs), leading to cyclooxygenase-2 (COX-2) induction and increased production of local thromboxane, and uric acid also upregulates the mRNA expression of platelet-derived growth factor A, C, and alpha receptors (81). Research has demonstrated that uric acid leads to impaired functioning of endothelial cells, potentially exacerbating the progression of diabetic neuropathy (73, 82). Thus, these potential pathological mechanisms suggest that elevated SUA levels are associated with an elevated likelihood of developing DPN. Two recent studies have reported that SUA levels vary among diabetes patients with and without neuropathy in the extremities, as well as those with and without sudomotor dysfunction (74).

The present meta-analysis indicated that there was a positive correlation between elevated HUA levels and an increased risk of DPN in individuals with T2DM, suggesting that uric acid may contribute to the development of DPN. The present meta-analysis also showed that the levels of SUA in patients with DPN were significantly higher compared to those without DPN, suggesting an association between elevated SUA levels and DPN in diabetes patients. Therefore, these findings suggested that an elevated concentration of uric acid in the bloodstream is associated with a higher likelihood of developing DPN.

Considering that SUA levels can be modified, interventions aimed at reducing uric acid may have potential preventive or therapeutic benefits for diabetic patients with DPN. At present, the clinical potential of uric acid-lowering interventions for DPN has been suggested by cross-section studies and case-control studies, highlighting the necessity for randomized controlled trials (RCTs) to test the efficacy of these interventions. Multi-center, blinded, randomized controlled trials, with strict inclusion and exclusion criteria, are needed. Unified uric acid determination and DPN detection, as well as DPN stage details, are required to eliminate multi-factor interference to objectively reflect the influence of uric acid-lowering intervention measures on DPN and provide new ideas for the prevention, diagnosis, and treatment of DPN.

### 4.1 Limitations and prospects

The present meta-analysis had several limitations. There is a crucial need for additional prospective cohort investigations to explore the impact of HUA on the susceptibility to DPN. While the present analysis included cross-sectional and case-control studies, conducting well-designed prospective cohort studies is essential for a more accurate assessment of the potential association between HUA and DPN risk. The present analysis focused solely on the correlation between HUA and DPN risk in patients with T2DM, as there was a lack of relevant research available for individuals diagnosed with T1DM. Therefore, further studies are necessary to evaluate this correlation, specifically in patients with T1DM.

Only one article in the literature analyzed the relationship between DPN severity and SUA levels (34). The results suggested that the prevalence of DPN decreases with increasing SUA levels, indicating that individuals with normal SUA levels may be at higher risk of developing DPN compared to those with lower SUA levels. However, the present analysis indicated that there were higher SUA levels in patients with DPN than those with mild DPN, suggesting the potential role of SUA in the progression or severity of DPN. Nonetheless, the exact mechanism underlying this relationship remains unclear and requires further investigation.

The present findings demonstrated that the effect of SUA levels on DPN patients varied depending on several factors, such as study location and sample size. For instance, a cross-sectional survey in China with a large sample size has suggested that high uric acid levels may reduce the risk of DPN (34). In contrast, a cross-sectional survey in Thailand has indicated that high uric acid levels are a risk factor for DPN (83). These discrepancies may reflect differences in lifestyle, genetic background, and healthcare systems among different regions. Moreover, studies with smaller samples showed larger effect sizes and higher heterogeneity, which may be due to greater instability and variability in the results of studies with small sample sizes. Conversely, studies with greater sample sizes showed smaller and non-significant effect sizes with reduced heterogeneity. These limitations emphasize the necessity for further prospective cohort studies and more rigorous study designs to mitigate heterogeneity and enhance the reliability of the findings.

### 4.2 Conclusion

In summary, the present study identified a correlation between increased SUA levels and an enhanced susceptibility to the development of DPN. Thus, high SUA may be a risk factor and potential predictor of DPN.

## Data availability statement

The original contributions presented in the study are included in the article/**Supplementary Material**. Further inquiries can be directed to the corresponding author.

## Author contributions

XL: Writing – original draft. XYZ: Writing – review & editing, Supervision, Conceptualization. XWZ: Writing – review & editing, Writing – original draft, Software, Methodology. XZ: Writing – review & editing, Visualization. GW: Writing – review & editing, Visualization. JS: Writing – review & editing, Validation. YY: Writing – review & editing, Investigation, Conceptualization. SF: Writing – review & editing, Conceptualization. JZ: Writing – review & editing, Validation. KZ: Writing – review & editing. JD: Writing – review & editing, Validation. JG: Writing – review & editing, Formal Analysis. WC: Writing – review & editing, Project administration.
