# Supplementary figures and images for: Association between serum uric acid levels and diabetic peripheral neuropathy in type 2 diabetes: a systematic review and meta-analysis
Source: Front Endocrinol (Lausanne). 2024 Jul 12;15:1416311. doi: 10.3389/fendo.2024.1416311 (PMC11272597; doi:10.3389/fendo.2024.1416311)

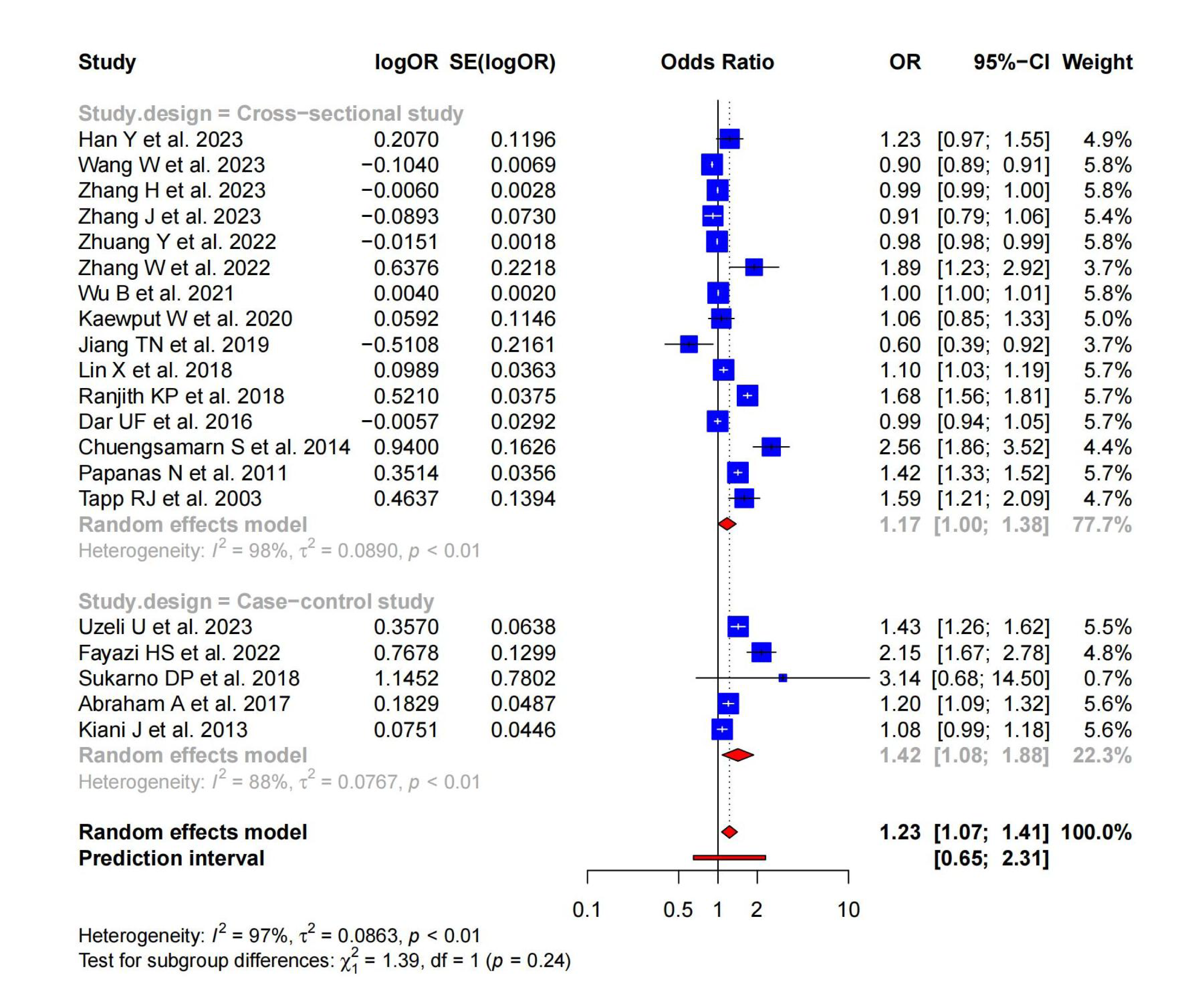

Supplement: Supplementary file 1 [file DataSheet_1.zip › Supplementary Material 3/Supplementary Material 3-1 Subgroup analysis by study type.tif]

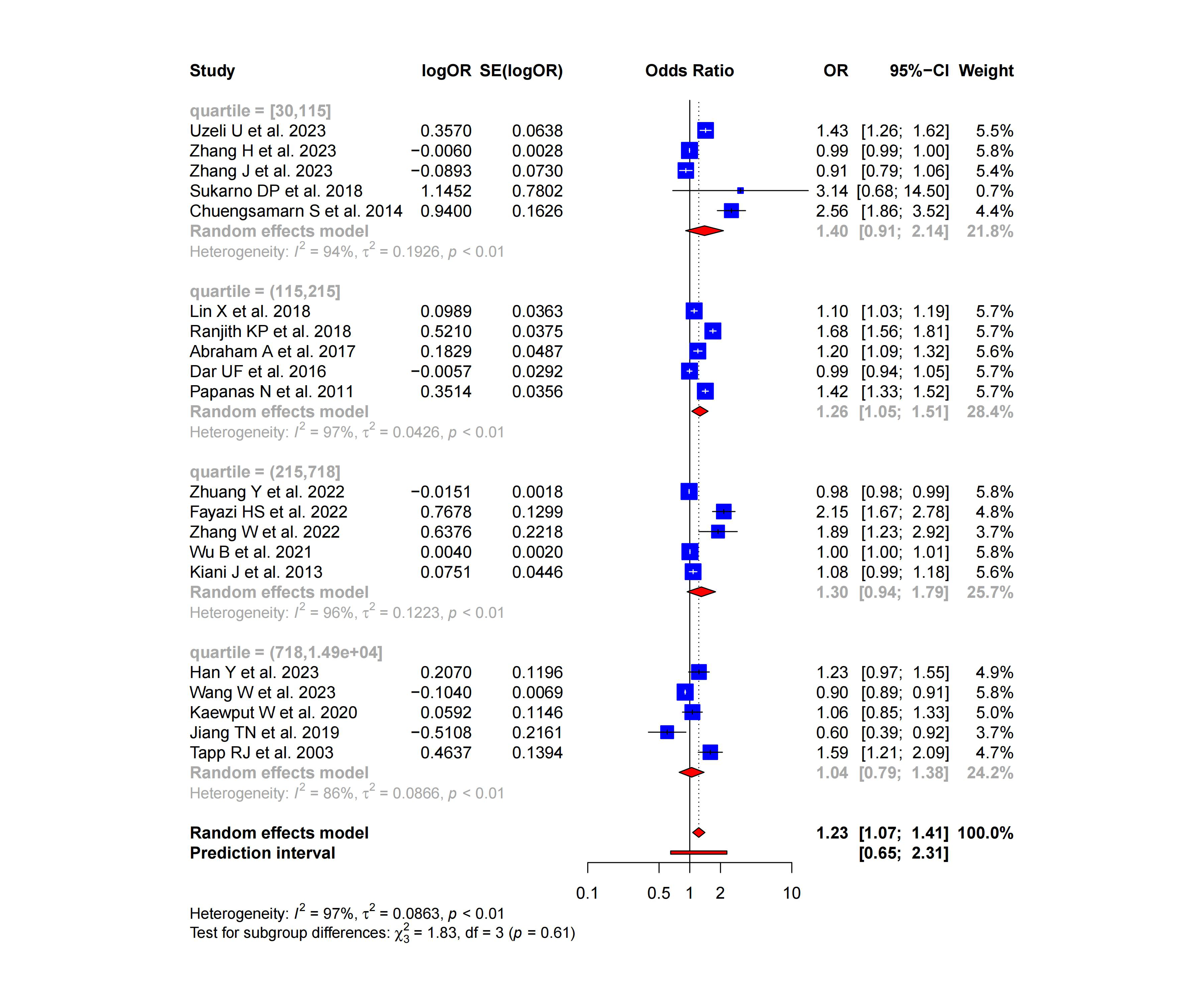

Supplement: Supplementary file 1 [file DataSheet_1.zip › Supplementary Material 3/Supplementary Material 3-3 Subgroup analysis by samplesize.tif]

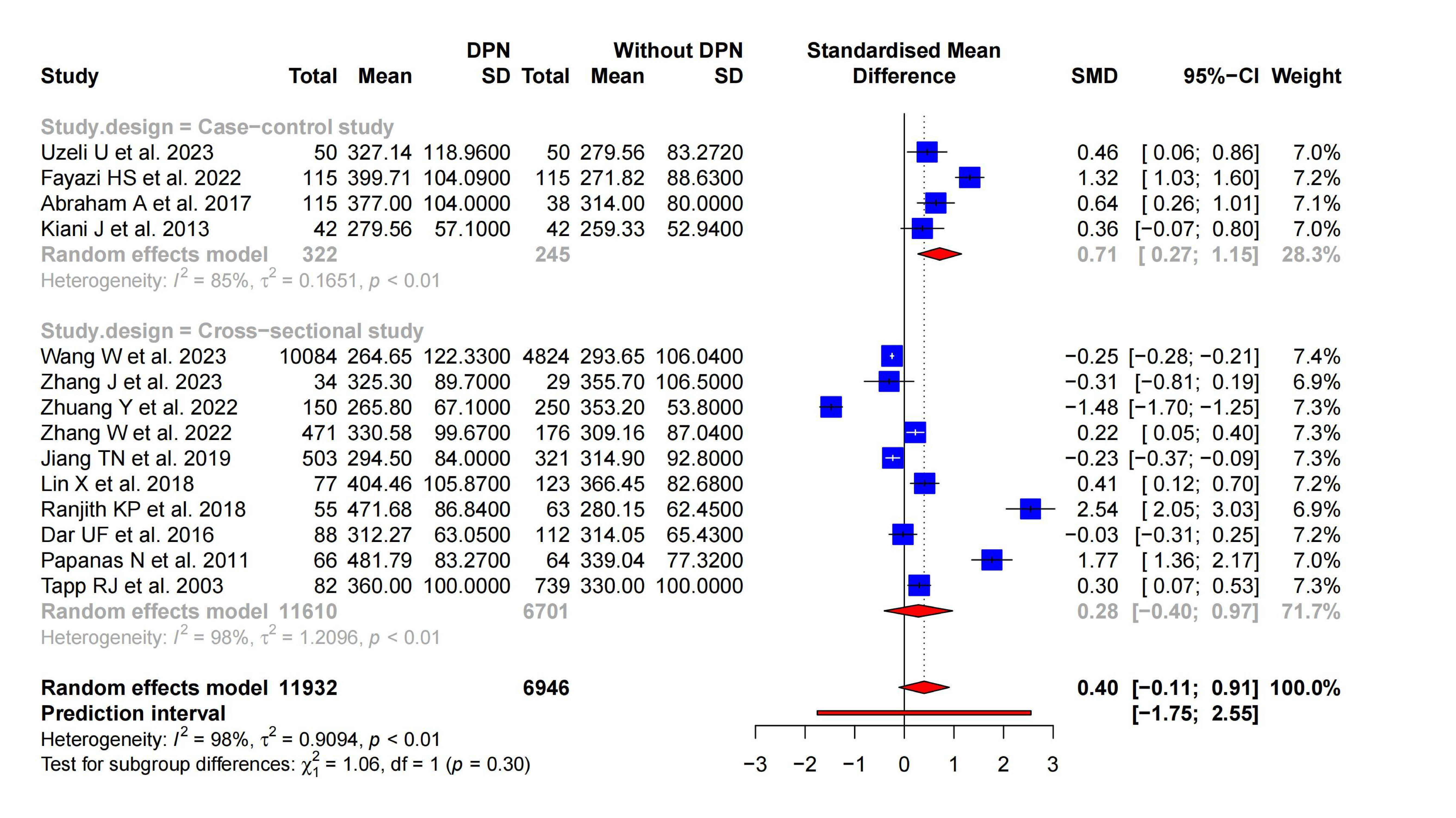

Supplement: Supplementary file 2 [file DataSheet_2.zip › Supplementary Material 5/Supplementary Material 5-1 Subgroup analysis by study type.tif]

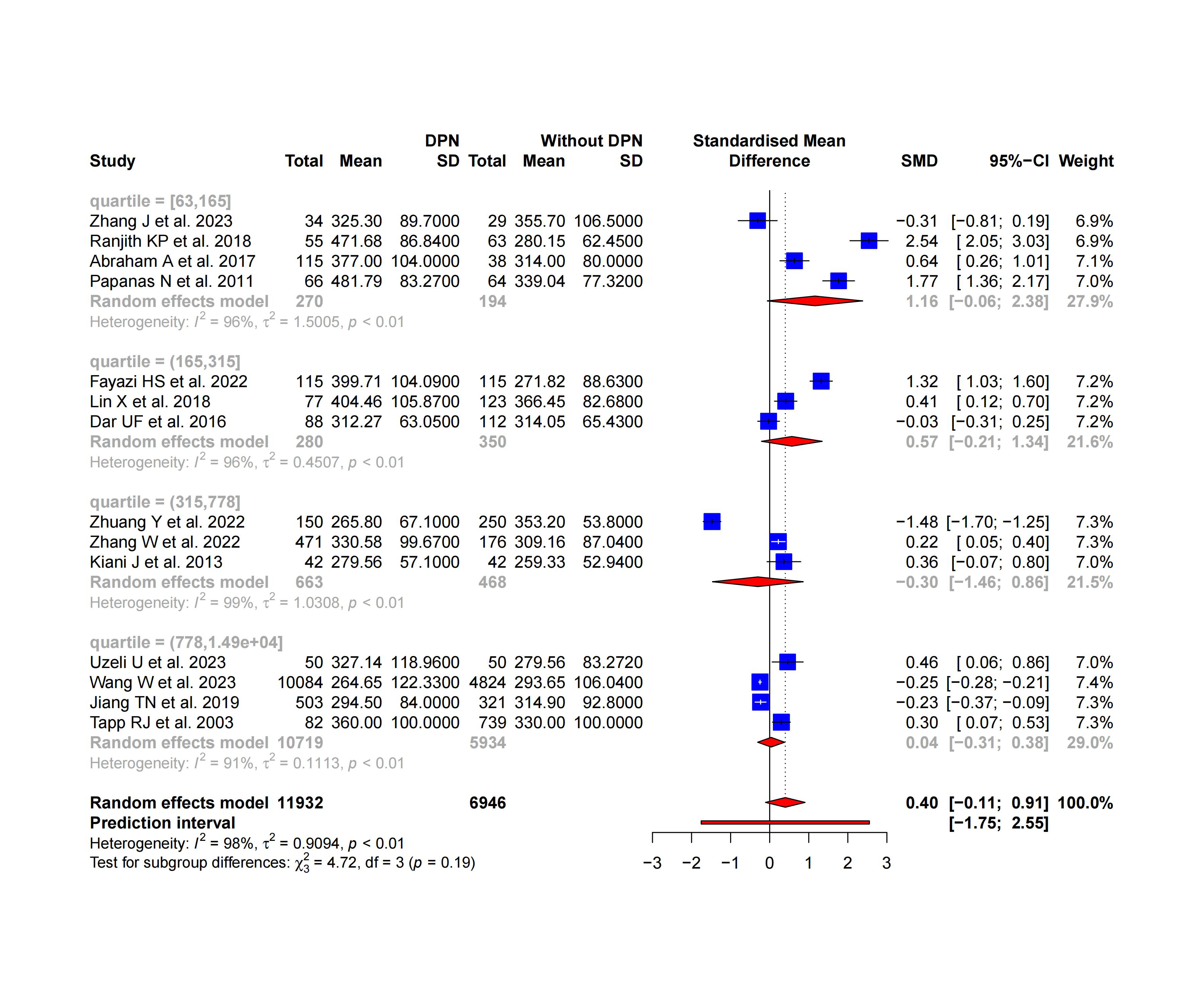

Supplement: Supplementary file 2 [file DataSheet_2.zip › Supplementary Material 5/Supplementary Material 5-3 Subgroup analysis by samplesize.tif]

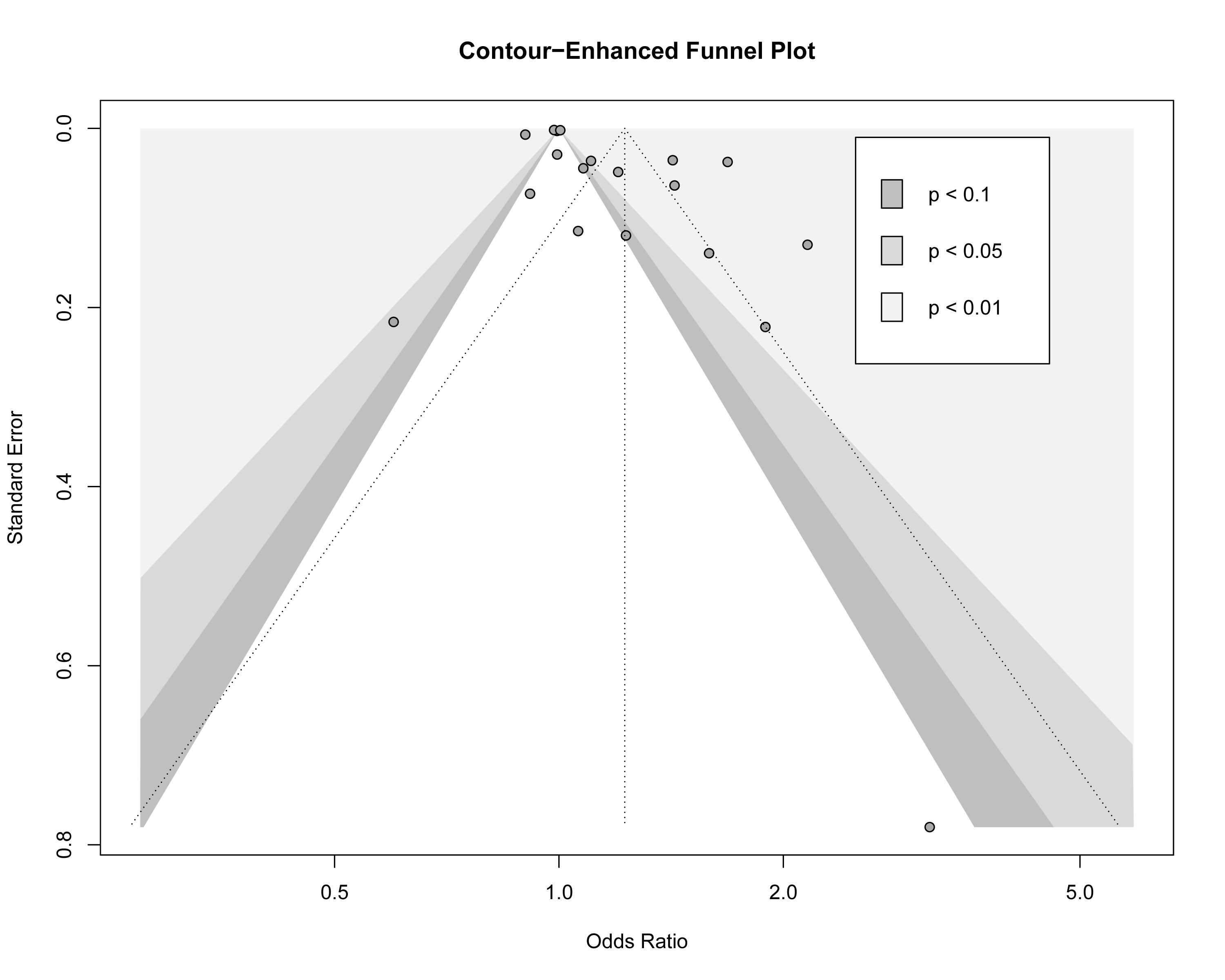

Supplement: Supplementary file 3 [file DataSheet_3.zip › Supplementary Material 6/Supplementary Material 6-1_Publication Bias_OR.tif]

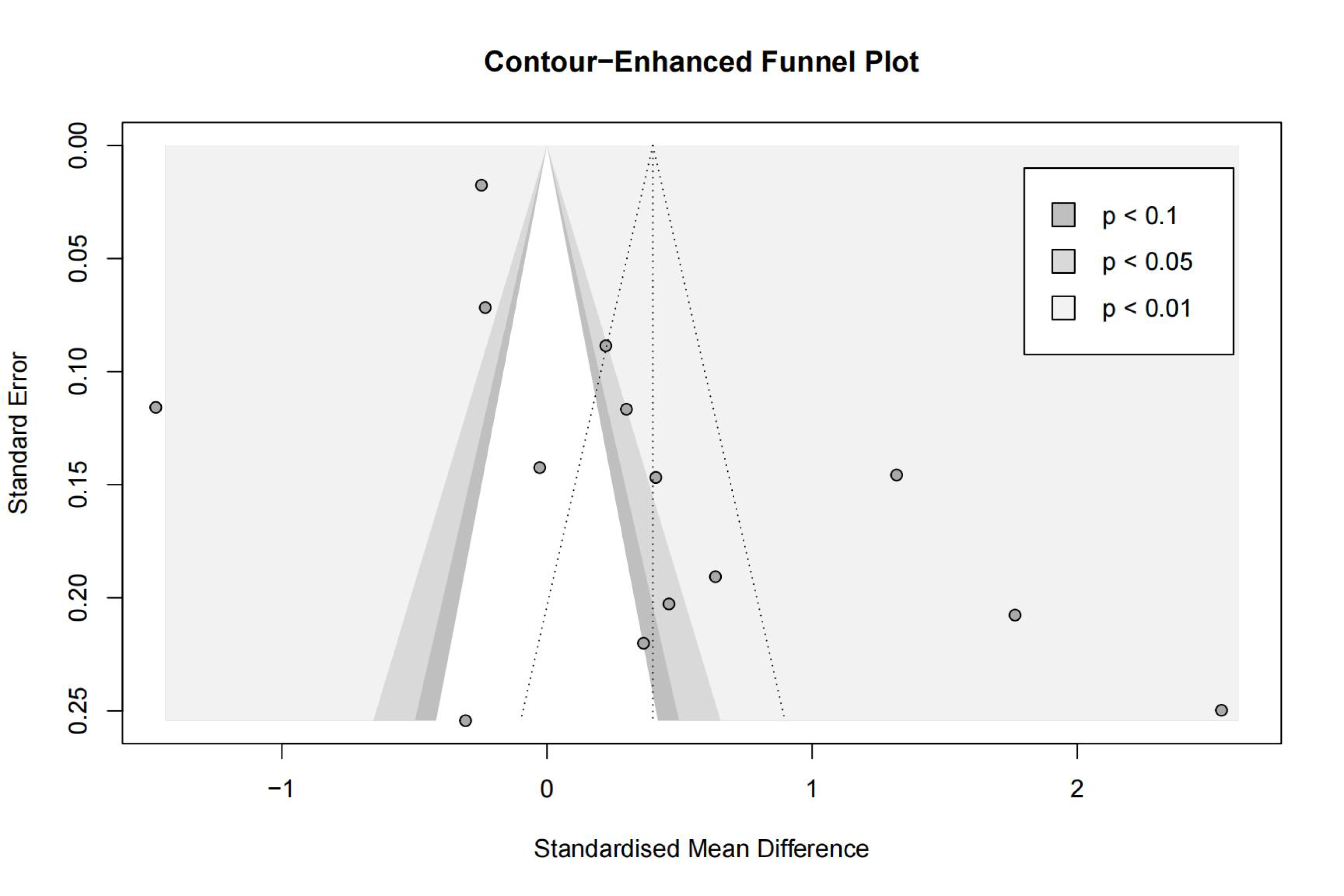

Supplement: Supplementary file 3 [file DataSheet_3.zip › Supplementary Material 6/Supplementary Material 6-2_Publication Bias_SMD.tif]

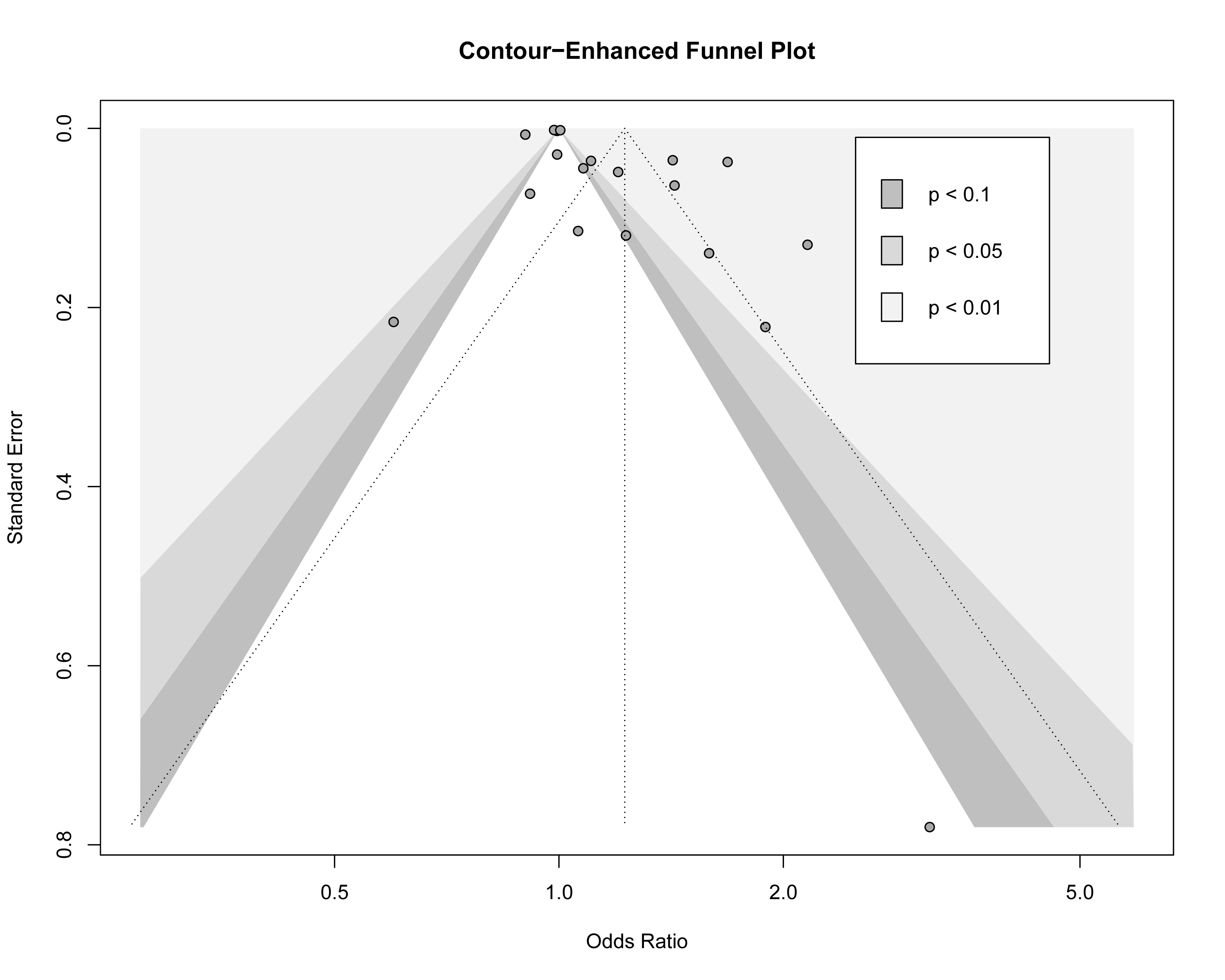

Supplement: Supplementary file 7 [file Image_1.tif]

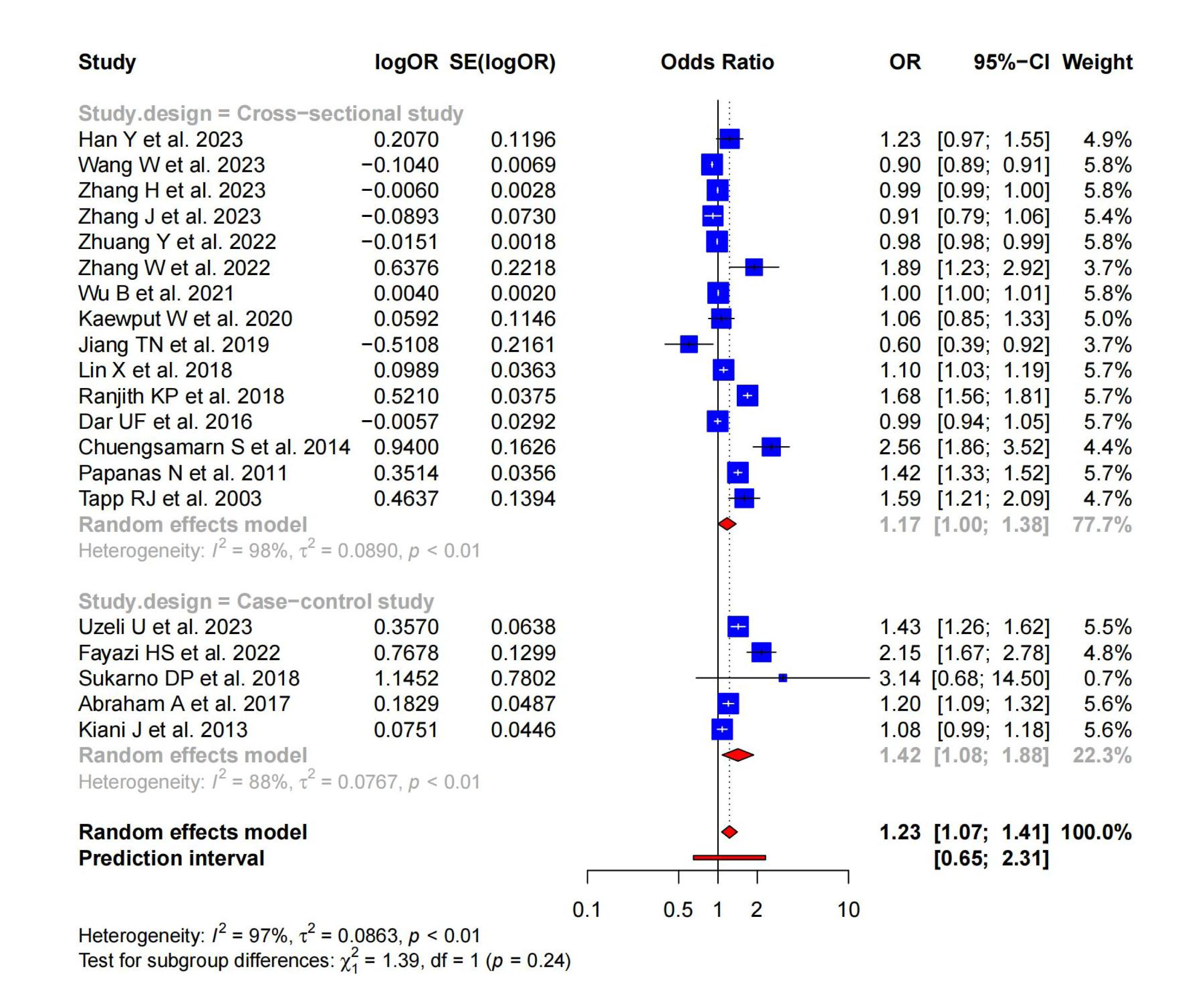

Supplement: Supplementary file 8 [file Image_2.tif]
