# Supplementary material for: Association between serum uric acid levels and diabetic peripheral neuropathy in type 2 diabetes: a systematic review and meta-analysis
Source: Front Endocrinol (Lausanne). 2024 Jul 12;15:1416311. doi: 10.3389/fendo.2024.1416311 (PMC11272597; doi:10.3389/fendo.2024.1416311)
Supplement: Supplementary file 10 [file Table_2.docx]

**Pubmed**

#1 "Gout"[Mesh]

#2 gout* OR tophus OR tophi OR tophaceous

#3 "Hyperuricemia"[Mesh]

#4 hyperuricemi* OR hyperuricaemi* OR hyperuricacid* OR elevated uric acid* OR high uric acid*

#5 #1 OR #2 OR #3 OR #4

#6 "Diabetes Mellitus"[Mesh]

#7 diabet*

#8 #6 OR #7

#9 "Peripheral Nervous System Diseases"[Mesh]

#10 neuropath* OR polyneuropath*

#11 #9 OR #10

#12 #8 AND #11

#13 #5 AND #12

**Embase**

('gout'/exp OR gout:ab,ti OR tophus:ab,ti OR tophi:ab,ti OR tophaceous:ab,ti OR 'hyperuricemia'/exp OR hyperuricemi*:ab,ti OR hyperuricaemi*:ab,ti OR hyperuricacid*:ab,ti OR 'elevated uric acid*':ab,ti OR 'high uric acid*':ab,ti) AND ('diabetes mellitus'/exp OR diabet*:ab,ti) AND ('peripheral neuropathy'/exp OR neuropath*:ab,ti OR polyneuropath*:ab,ti)

**Web of Science**

(TS=(gout) OR TS=(tophus) OR TS=(tophi) OR TS=(tophaceous) OR TS=(hyperuricemi*) OR TS=(hyperuricaemi*) OR TS=(hyperuricacid*) OR TS=(elevated uric acid*) OR TS=(high uric acid*)) AND (TS=(diabetes mellitus) OR TS=(diabet*)) AND (TS=(peripheral nervous system diseases) OR TS=(neuropath* ) OR TS=(polyneuropath*))

**Cochrane Library：**

#1 MeSH descriptor: [Gout] explode all trees

#2 (gout*):ti,ab,kw OR (tophus):ti,ab,kw OR (tophi):ti,ab,kw OR (tophaceous):ti,ab,kw

#3 MeSH descriptor: [Hyperuricemia] explode all trees

#4 (hyperuricemi*):ti,ab,kw OR (hyperuricaemi*):ti,ab,kw OR (hyperuricacid*):ti,ab,kw OR (elevated uric acid*):ti,ab,kw OR (high uric acid*):ti,ab,kw

#5 #1 OR #2 OR #3 OR #4

#6 MeSH descriptor: [Diabetes Mellitus] explode all trees

#7 (diabet*):ti,ab,kw

#8 #6 OR #7

#9 MeSH descriptor: [Peripheral Nervous System Diseases] explode all trees

#10 (neuropath*):ti,ab,kw OR (polyneuropath*):ti,ab,kw

#11 #9 OR #10

#12 #8 AND #11

#13 #5 AND #12
